# Routine clinical laboratory evaluation demonstrates limited utility of Fourier-transform infrared spectroscopy for subspecies identification of the *Mycobacterium abscessus* complex

**DOI:** 10.1128/jcm.01087-26

**Published:** 2026-10-12

**Authors:** Mitsunori Yoshida, Kohei Doke, Takeshi Komine, Hanako Fukano, Azumi Fujinaga, Yoshihiko Hoshino

**Affiliations:** 1 Leprosy Research Center, National Institute of Infectious Diseases, Japan Institute for Health Security 231182, Tokyo, Japan; 2 Bruker Japan K.K., Kanagawa, Japan; The University of North Carolina at Chapel Hill School of Medicine, Chapel Hill, North Carolina, USA

**Keywords:** clinical microbiology, subspecies identification, IR Biotyper, Fourier-transform infrared spectroscopy, *Mycobacterium abscessus* complex

## LETTER

The *Mycobacterium abscessus* complex (MABC) comprises three closely related subspecies whose accurate identification is clinically important because inducible macrolide resistance is largely determined by the functional status of *erm*(41) (1). However, reliable subspecies discrimination remains challenging in routine clinical microbiology.

Matrix-assisted laser desorption/ionization time-of-flight mass spectrometry (MALDI-TOF MS) has improved species-level identification of non-tuberculous mycobacteria but remains unreliable for MABC subspecies discrimination. Although machine learning–based approaches have shown improved performance, they require customized analytical pipelines that limit routine implementation (2). Likewise, lipid fingerprinting using the MALDI Biotyper Sirius failed to reliably distinguish MABC subspecies (3). Fourier-transform infrared (FTIR) spectroscopy is a rapid phenotypic typing platform (4), and one previous study reported successful differentiation of *M. abscessus* subsp. *abscessus* and subsp. *massiliense* under optimized culture conditions, particularly on Löwenstein–Jensen medium (5).

We evaluated whether FTIR could discriminate MABC subspecies under routine clinical laboratory conditions. A total of 137 clinical and environmental isolates, together with three reference strains (140 isolates total), previously assigned by whole-genome sequencing, were analyzed (Table S1). FTIR spectra were obtained from liquid cultures using the IR Biotyper and analyzed as described in the supplemental methods. Broad spectral regions and targeted spectral windows generated using multiple software-defined wavenumber thresholds (35%–85%) were evaluated by principal component analysis (PCA) and supervised machine-learning classification.

**Fig 1:**
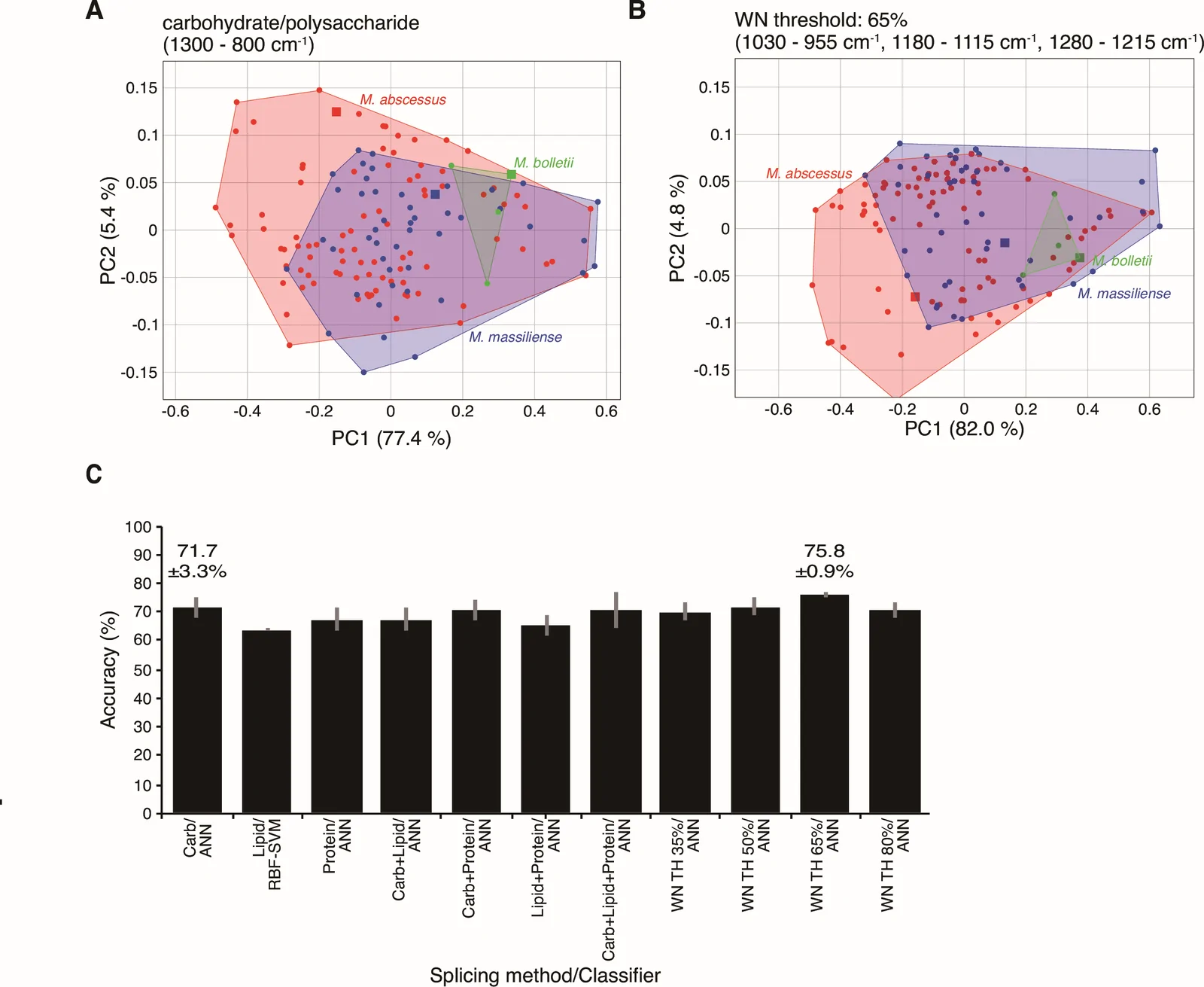
FTIR spectroscopy fails to provide robust subspecies-level discrimination within the *Mycobacterium abscessus* complex. (**A**) Representative principal-component analysis (PCA) score plot of whole-cell FTIR spectra from 137 clinical and environmental isolates assigned to subspecies by whole-genome sequencing and three reference strains (Table S1). Broad spectral categories corresponding to carbohydrate/polysaccharide (1,300–800 cm^−1^)-, protein/amide (1,800–1500 cm^−1^)-, and lipid (3,000–2,800 cm^−1^)-associated regions were evaluated individually and in combination. The representative plot shown here illustrates the substantial overlap among *M. abscessus*, *M. massiliense*, and *Mycobacterium bolletii* observed across all tested broad spectral profiles; additional analyses are shown in Fig. S1. (**B**) PCA score plot of narrower candidate spectral windows generated using wavenumber (WN) thresholds of 35%, 50%, 65%, and 80% to identify regions with potential discriminatory value. All selected windows were localized within the carbohydrate/polysaccharide (1,300–800 cm^−1^)-associated spectral range (see Fig. S2). The corresponding PCA plots obtained at WN thresholds of 35%, 50%, and 80% are also shown in Fig. S2. (**C**) Classification performance of supervised machine learning models applied to the selected candidate spectral windows. Three algorithms were evaluated: linear support vector machine (Linear-SVM), support vector machine with a radial basis function kernel (RBF-SVM), and artificial neural network (ANN). Bars indicate mean cross-validation accuracy. Maximum mean accuracy was 75.8%.

FTIR consistently failed to discriminate the three MABC subspecies. No broad spectral region enabled separation either individually or in combination. PCA demonstrated substantial overlap among the three subspecies across all spectral profiles (Fig. 1A; Fig. S1). The selected windows consistently mapped to three carbohydrate-associated subregions, but none improved PCA discrimination (Fig. 1B; Fig. S2). Supervised machine learning classification likewise showed limited performance, with a maximum mean cross-validated accuracy of only 75.8%, insufficient for reliable clinical identification (Fig. 1C; Fig. S3). PCA also failed to separate isolates according to colony morphotype or *erm*(41) T28C status (Fig. S4).

These findings demonstrate that FTIR-derived spectral information is insufficient for routine subspecies identification of MABC. Unlike previous proof-of-concept studies performed under optimized culture conditions (5), our study evaluated FTIR using liquid cultures in a workflow reflecting routine clinical laboratory practice. The inability of both targeted spectral-window selection and supervised classifiers to improve discrimination suggests that the limitation is not simply analytical. Rather, the close genetic relatedness of the three subspecies, together with substantial within-subspecies diversity and biological variability, likely obscures reproducible spectroscopic signatures (6). These findings further suggest that improvements in computational analyses alone may be insufficient to overcome the current limitations of the FTIR workflow. Instead, advances in sample preparation, culture standardization, or spectral acquisition may be required before FTIR can achieve clinically useful subspecies discrimination. Our conclusions are strengthened by evaluation of one of the largest whole-genome-sequenced MABC collections examined by FTIR to date. Collectively, these findings indicate that the current FTIR workflow does not provide sufficient discriminatory power for routine MABC subspecies identification. Until further methodological improvements become available, molecular methods remain the reference standard for definitive subspecies assignment.

## SUPPLEMENTAL MATERIAL

Supplemental materials jcm.01087-26-s0001.docx Supplemental methods and Fig. S1 to S4.

Table S1 jcm.01087-26-s0002.xlsx Strains used in this study.

ASM does not own the copyrights to Supplemental Material that may be linked to, or accessed through, an article. The authors have granted ASM a non-exclusive, world-wide license to publish the Supplemental Material files. Please contact the corresponding author directly for reuse.
