# Supplementary material for: Routine clinical laboratory evaluation demonstrates limited utility of Fourier-transform infrared spectroscopy for subspecies identification of the Mycobacterium abscessus complex
Source: J Clin Microbiol. 2026 Oct 12;64(11):e01087-26. doi: 10.1128/jcm.01087-26 (PMC13753746; doi:10.1128/jcm.01087-26)
Supplement: Supplementary file 1 [file jcm.01087-26-s0001.docx]

***Supplemental material***

**Routine Clinical Laboratory Evaluation Demonstrates Limited Utility of Fourier-Transform Infrared Spectroscopy for Subspecies Identification of the *Mycobacterium abscessus* Complex**

Mitsunori Yoshida, Kohei Doke, Takeshi Komine, Hanako Fukano, Azumi Fujinaga, Yoshihiko Hoshino

**Supplemental Methods**

**Bacterial isolates**

A total of 140 *Mycobacterium abscessus* complex (MABC) strains, including clinical isolates, environmental isolates, and three reference strains, were included in this study (Table S1). Clinical and environmental isolates were assigned to subspecies by whole-genome sequencing as previously described (1). Reference strains, *M. abscessus* subsp. *abscessus* JCM 13569 (=ATCC 19977), *M. abscessus* subsp. *massiliense* JCM 15300, and *M. abscessus* subsp. *bolletii* JCM 15297 (=BD), were obtained from the Japan Collection of Microorganisms, RIKEN BioResource Research Center (BRC-JCM; Ibaraki, Japan). Before FTIR analysis, all strains were cultured under standardized conditions to minimize technical variation among samples. Frozen stocks were subcultured on Middlebrook 7H10 agar supplemented with 10% oleic acid-albumin-dextrose-catalase (OADC) at 30°C. A single colony from each strain was inoculated into Middlebrook 7H9 broth supplemented with 10% OADC and cultured at 30°C for 3 to 5 days before FTIR analysis.

**Sample preparation and FTIR spectra acquisition using the IR Biotyper**

FTIR spectra were acquired using the IR Biotyper system (Bruker, Germany) according to the manufacturer’s instructions, with minor modifications. For instrument validation, each kit-provided IR test standard (IRTS1 and IRTS2) was reconstituted with 90 μL of sterile water, incubated for 15 min at room temperature, mixed gently, and supplemented with 90 μL of ethanol. For each run, at least two replicate spots of each standard were applied to the silicon microtiter plate and dried together with the study samples. For sample preparation, bacterial biomass recovered from liquid culture was suspended in 50 μL of 70% ethanol in the sample tube provided with the kit and mixed thoroughly by vortexing. Subsequently, 50 μL of sterile water was added, and the suspension was vortexed again. Aliquots (15 μL) were spotted onto the silicon microtiter plate in quadruplicate and allowed to dry for 20 to 30 min at room temperature before measurement. Spectra were acquired independently from the four replicate spots. The four technical replicate spectra were averaged to generate a single mean spectrum for each strain, which was used for all subsequent analyses.

**Data processing and analysis**

Spectra were analyzed using OPUS version 8.2 and IR Biotyper Client version 7.5 (Bruker, Germany) as described elsewhere with some modifications (2). Broad spectral regions associated with carbohydrates/polysaccharides (1300 to 800 cm^-1^), proteins/amides (1800 to 1500 cm^-1^), and lipids (3000 to 2800 cm^-1^) were evaluated individually and in combination. Principal-component analysis (PCA) was used as an initial unsupervised approach to assess clustering of *M. abscessus* subsp. *abscessus*, subsp. *massiliense*, and subsp. *bolletii*. To identify potentially informative narrower spectral windows, the software-defined WN threshold was varied from 35% to 80% in 15% increments (35%, 50%, 65%, and 80%). Candidate regions selected by this procedure were further evaluated by PCA. Supervised machine-learning classification was applied to each broad spectral region, all combinations of the broad regions, and the candidate windows selected at each WN threshold using three algorithms: linear support vector machine (Linear-SVM), support vector machine with a radial basis function kernel (RBF-SVM), and artificial neural network (ANN). Accuracy was calculated as the mean proportion of correctly classified isolates across cross-validation folds. In a supplementary analysis, *M. abscessus* isolates were also evaluated according to *erm*(41) T28C status. Models were evaluated using stratified 4-fold cross-validation repeated 5 times. All replicate spectra from the same isolate were assigned to the same fold. Accuracy was calculated for each fold as the number of correctly classified isolates divided by the total number of isolates, and the mean and standard deviation across folds were reported. In supplementary analyses, isolates were stratified according to colony morphotype (rough, smooth, or mixed), as recorded on Middlebrook 7H10 agar, and evaluated by PCA using the carbohydrate/polysaccharide-associated region and the candidate windows selected at a WN threshold of 65%. *M. abscessus* subsp. *abscessus* isolates were also evaluated according to *erm*(41) T28 or T28C status.

**Supplemental Table Legend**

**Table S1.** Characteristics of the *Mycobacterium abscessus* complex isolates included in this study. Clinical and environmental isolates and reference strains used for FTIR analysis are listed. Subspecies assignments of clinical and environmental isolates were determined by whole-genome sequencing as previously described (1).

**Supplemental Figure Legends**

**Figure S1**

Additional PCA plots of broad FTIR spectral regions evaluated for subspecies discrimination within the MABC. PCA score plots for the broad FTIR spectral categories evaluated in this study, including carbohydrate/polysaccharide-, protein/amide-, and lipid-associated regions, analyzed individually and in combination. Across all tested spectral profiles, substantial overlap was observed among *M. abscessus*, *M. massiliense*, and *M. bolletii*, indicating that no broad spectral region or combination of regions enabled clear subspecies-level separation. Dots represent individual isolates, colored polygons indicate convex hulls for each subspecies, and colored squares indicate the corresponding reference strains (*M. abscessus* ATCC 19977, *M. massiliense* JCM 15300, and *M. bolletii* BD). Subspecies assignments were defined by whole-genome sequencing as described previously.

**Figure S2**

PCA plots of candidate FTIR spectral windows selected by varying the software-defined WN threshold. PCA score plots for the candidate spectral windows selected by varying the software-defined WN threshold from 35% to 80% to identify narrower regions with potential discriminatory value. All selected windows were localized within subregions of the carbohydrate/polysaccharide-associated spectral range. However, none of the selected windows yielded clear separation of *M. abscessus*, *M. massiliense*, and *M. bolletii*, indicating that refinement of the spectral window did not improve subspecies discrimination. Dots represent individual isolates, colored polygons indicate convex hulls for each subspecies, and colored squares indicate the corresponding reference strains (*M. abscessus* ATCC 19977, *M. massiliense* JCM 15300, and *M. bolletii* BD).

**Figure S3**

Cross-validated classification accuracy of supervised machine-learning models for subspecies identification within the *Mycobacterium abscessus* complex. Classification performance was evaluated using three supervised machine-learning algorithms: linear support vector machine (Linear-SVM), support vector machine with a radial basis function kernel (RBF-SVM), and artificial neural network (ANN). Models were applied to the carbohydrate/polysaccharide-associated (Carb), lipid-associated, and protein/amide-associated spectral regions individually and in all possible combinations, as well as to candidate spectral windows selected using software-defined WN thresholds of 35%, 50%, 65%, and 80%. Whole-genome sequencing-based subspecies assignments were used as class labels. For each cross-validation fold, accuracy was calculated as the number of correctly classified isolates divided by the total number of isolates, and bars represent the mean accuracy across folds. Error bars indicate the variability across cross-validation folds. WN TH, wavenumber threshold.

**Figure S4** Principal-component analysis of FTIR spectra according to colony morphotype and *erm*(41) T28C status. (A and B) PCA score plots of *Mycobacterium abscessus* complex isolates stratified by colony morphotype as rough, smooth, or mixed. Spectra were analyzed using the broad carbohydrate/polysaccharide-associated region (1,300 to 800 cm⁻¹) (A) or three narrower candidate windows selected at a software-defined WN threshold of 65% (1,030 to 955, 1,180 to 1,115, and 1,280 to 1,215 cm⁻¹) (B). (C and D) PCA score plots of *M. abscessus* subsp. *abscessus* isolates stratified according to *erm*(41) T28 or T28C status. Spectra were analyzed using the broad carbohydrate/polysaccharide-associated region (C) or the three candidate windows selected at a WN threshold of 65% (D). Neither colony morphotype nor *erm*(41) T28C status resulted in clear separation of the corresponding isolate groups. Dots represent individual isolates, colored polygons indicate the convex hulls of each group, and square symbols indicate spectra obtained from reference strains, where applicable.

**Supplemental Figures**

**Figure S1**

**Figure S2**

**Figure S3**

**Figure S4**

**Supplemental References**

1. Yoshida M, Sano S, Chien J-Y, Fukano H, Suzuki M, Asakura T, Morimoto K, Murase Y, Miyamoto S, Kurashima A, Hasegawa N, Hsueh P-R, Mitarai S, Ato M, Hoshino Y. 2021. A novel DNA chromatography method to discriminate Mycobacterium abscessus subspecies and macrolide susceptibility. EBioMedicine 103187.
2. Nakajima N, Jinnai M, Izumiyama S, Kuroki T. 2024. Evaluating Fourier-transform infrared spectroscopy with IR Biotyper as a faster and simpler method for investigating the sources of an outbreak of legionellosis. Eur J Clin Microbiol Infect Dis 43(5):991-997.
